# Alternative Means of Informed Consent in Cardiology: Strategies and Effectiveness in a Group of Italian Patients

**DOI:** 10.3390/bs13050430

**Published:** 2023-05-19

**Authors:** Ines Testoni, Lucia Ronconi, Francesca Lampis, Erika Iacona, Josephine Zammarrelli, Sara Pompele, Roberto Valle, Gabriele Boscolo, Diego De Leo

**Affiliations:** 1 Department of Philosophy, Sociology, Pedagogy and Applied Psychology (FISPPA), University of Padova, 35122 Padova, Italy; ines.testoni@unipd.it (I.T.); francesca.lampis7@gmail.com (F.L.); erika.iacona@unipd.it (E.I.); sara.pompele@unipd.it (S.P.); 2 Emili Sagol Creative Arts Therapies Research Center, University of Haifa, Haifa 3498838, Israel; 3 IT and Statistical Services, Multifunctional Centre of Psychology, University of Padova, 35122 Padova, Italy; l.ronconi@unipd.it; 4 Human Rights Centre Antonio Papisca, University of Padua, 35137 Padova, Italy; 5 De Leo Fund, 35137 Padua, Italy; jzammarrelli@virgilio.it; 6 Department of Cardiology, General Hospital, 30015 Chioggia, Italy; roberto.valle@aulss3.veneto.it (R.V.); gabriele.boscolo@aulss3.veneto.it (G.B.); 7 Slovene Centre for Suicide Research, Primorska University, 6000 Koper, Slovenia; 8 Australian Institute for Suicide Research and Prevention, Griffith University, Brisbane, QLD 4122, Australia

**Keywords:** written informed consent, coronary angiography, patient-centred care, multi-media informed consent

## Abstract

Informed consent practices in healthcare represent a fundamental element of patient-centred care; however, the traditional use of a written, paper-based description of the medical procedure to obtain informed consent presents many limitations. This research aimed to evaluate the effects of an alternative modality of obtaining informed consent using a brief informative video for patients waiting to undergo a coronary angiography procedure in Italy. The study involved 40 participants—28 males and 12 females (mean age: 68.55, SD = 13.03)—divided equally into two groups: one group received the video-based informed consent and the other received a traditional paper-based form. Each group was asked to fill in two questionnaires; one was created by the researchers to measure the patient’s level of understanding of the given information and the perception of usefulness of the informed consent, and the other was the Depression Anxiety Stress Scales-21 (DASS-21), which evaluates levels of anxiety, depression and stress. A comparison of the results of the two groups showed that video-based informed consent allowed participants to better understand the given information, to feel more confident concerning their subjective comprehension of it and to perceive the video-based informed consent as more useful than the traditional one. The video-based informed consent did not lead to higher levels of anxiety, depression or stress among the participants. It can be hypothesized that video-based formats may represent a more useful, understandable and safe alternative to traditional paper-based informed consent in healthcare.

## 1. Introduction

### 1.1. Informed Consent in Medicine

Informed consent in medicine represents a step of fundamental importance in the process of properly taking care of patients, since it allows patients to effectively become the protagonists of their clinical history and to access diagnostic tests and treatments in a fully aware and prepared way [1].

It also represents a crucial step in the transition from an older, paternalistic conception of medicine in which doctors took upon themselves the burden of care and in which information was provided to the patient exclusively in the modality and at the time deemed appropriate by the doctors themselves to a more patient-centred approach in which there is an equal exchange between healthcare professionals and the patients who make a valuable contribution to the therapeutic relationship [2].

The latter aspect of this new model of healthcare has yet to be fully achieved, with numerous situations in healthcare—particularly involving complex conditions linked to serious and/or terminal diseases—in which healthcare professionals choose not to fully inform patients for fear of upsetting or scaring them, thus renouncing the good practices of truth-telling [3].

Although some healthcare professionals have expressed doubts over the value of informed consent practices, stirring a debate concerning the potential risks of providing accurate information to patients for fear of increasing their distress [2], many studies have shown that the provision of adequate and precise information before a healthcare intervention has a positive effect on a patient’s experience, increasing their satisfaction with the care decision and the level of care received, as well as decreasing their levels of anxiety, especially before invasive procedures such as surgery [4,5].

This makes it evident that there is a fundamental need to provide precise information to patients and to collect proper informed consent.

In Italy, the fundamental necessity to obtain patients’ informed consent for every healthcare procedure was established in article no. 32 of the Constitution of the Italian Republic, which states that the protection of each person’s health is fundamental with due regard for individual freedom. Furthermore, a recent law of great value in the Italian context has more precisely regulated the matter of informed consent. This is Law no. 219/2017 [6], which, in its article no. 1, states how, in compliance with the principles of the Constitution and the Charter of Fundamental Rights of the European Union, the right to self-determination in healthcare is fundamental, establishing that no healthcare treatment can be started or continued without the patient’s freely given informed consent. It also makes it clear that patients have the right to full awareness of their health condition and to be informed of the benefits and risks of diagnostic tests and treatments, of any alternative healthcare interventions and of the possible consequences of refusal of a healthcare treatment or diagnostic assessments [6].

### 1.2. Limits in the Implementation of Traditional Informed Consent and Current Alternatives

Despite the importance of informed consent, procedures for procuring it often present shortcomings in their implementation. Numerous studies, both internationally and in Italy, have found that the traditional model of informed consent, consisting of a written document of several pages containing all the information the patient might need and which the person must read and sign, can end up being inaccessible to a large part of the population, as such forms often consist of complex and specialized terminology, leading to a signature that does not truly indicate a precise understanding of what has been read [7,8].

Another shortcoming that has been reported over the years is that sometimes the information provided is not enough to ensure the person has a good understanding of the procedures that will be implemented, an aspect to which other obstacles are sometimes added, such as little time available for patients to fully read the information sheet and for healthcare personnel to answer any questions that might arise, as well as poor training for the personnel themselves concerning the most appropriate ways of providing information [9,10,11].

To address these limitations, new modalities for presenting information to patients and for obtaining their informed consent have been introduced. In the surgical field, for example, different formats have appeared, often accompanied by images and graphic narrations that can be easily understood by patients [12,13]. These can include various multi-media methods, such as informative videos that present the necessary information in an active and engaging way [14,15].

The studies carried out in this area have highlighted how these formats, particularly informed consent presented through short informative videos, are generally appreciated more by patients, who report greater satisfaction with the information provided and greater understanding and recall of what is communicated through this medium compared to traditional paper-based informed consent [16,17,18].

### 1.3. Informed Consent in Cardiology

Cardiology is among the healthcare fields in which informed consent plays a particularly preponderant role, given the high prevalence of related pathologies and that cardiovascular disease (CVD), particularly coronary artery disease (CAD), is one of the most common causes of mortality worldwide [19]. In Italy, cardiovascular diseases are the leading cause of death, accounting for 34.8% of all deaths [20].

Many studies have reported the severe burden presented by cardiovascular and coronary diseases in terms of obstacles to the patient’s social relationships, work and quality of life [21] as well as worsening psychological well-being expressed by anxiety, depressed mood and even symptoms of post-traumatic stress [22].

Some diagnostic procedures used in cardiology can themselves represent an element of intense psycho-physical distress for the patient, particularly ones that involve surgery, such as coronary angiography, which is an invasive diagnostic procedure that allows precise visualization of the patient’s coronary arteries through a probe introduced by the arterial route, usually through the femoral artery [23].

It has been revealed by some studies that even merely knowing that one has to undergo this type of diagnostic test can cause significant levels of anticipatory anxiety and concern in relation to the invasiveness of the procedure, its course and the possibility of post-surgery pain [24,25].

Such distress can also be increased by poor or insufficient information being provided to the patient about the precise aspects of the procedure [26], an element that reiterates the fundamental importance of providing adequately informed consent prior to any medical procedure, especially if it is highly invasive.

Unfortunately, the same limits of traditional informed consent procedures have been found in the field of cardiology, particularly for informed consent to coronary angiography, with studies worldwide reporting poor effectiveness of written informed consent procedures, little satisfaction on the part of patients and poor understanding of the information provided, especially by people who lack knowledge of the healthcare field and its terminology [27,28,29,30].

Some studies have considered the wishes of cardiology patients waiting to undergo cardiac surgery in terms of desire for information, with a consensus that a significant majority of patients prefer to receive detailed and precise information about the operation, its risks and the postoperative course [31].

Therefore, even in the specific field of cardiology, multi-media methods for the presentation of informed consent have been tested, confirming that these alternative tools, particularly those in the form of short informative videos, are able to improve the knowledge acquired by the patient and the degree of satisfaction they have with the information received [32,33,34,35].

Some studies focusing on coronary angiography have found video information procedures to also have a positive effect on the patient’s level of anxiety, blood pressure and heart rate [36,37,38].

Very few studies on multi-media informed consent for coronary angiography have been conducted in the Italian context. However, the ones that can be found in the literature confirm that video-based informed consent is a more efficacious alternative to the traditional paper-based informed consent [39,40].

The present research is in this context and has the aim of further exploring the issue of the effectiveness of multi-media video formats of informed consent in cardiology, specifically with coronary angiography procedures for which very few specific studies are yet available in Italy, in order to evaluate the possible differences in impact on patients’ experiences compared to more traditional informed consent modalities.

## 2. Objectives

As previously mentioned, the general aim of the study was to verify whether the use of video-based informed consent has an impact on patients who are undergoing invasive medical procedures (specifically coronary angiography) that is different from that of the more traditional paper-based written informed consent.

This general aim was divided into more specific objectives, as follows:

1. To verify patients’ objective understandings of the information received by comparing the results of questionnaires given to the two groups—one whose members were given a traditional paper-based informed consent (control group) and one whose members were given a video-based informed consent (experimental group);
2. To analyse the sample’s perceptions of their own subjective understandings of the information contained in the informed consent they received, verifying the presence of any differences between the experimental and the control group;
3. To verify the perception of usefulness of the two different kinds of informed consent by the participants in both groups;
4. To analyse the variables of anxiety, stress and depression in the two groups to assess whether video-based informed consent, since it presents information in a more direct and explicit way, has a different impact on patients’ psychological experiences than traditional paper-based consent.

## 3. Materials and Methods

### 3.1. Participants

The research was conducted in the cardiology ward of a hospital in the north-east of Italy. In order to proceed with recruitment, a meeting was arranged with the directors of the cardiology department and the healthcare personnel of the ward in order to accurately illustrate the research objectives, methodology and procedure. The healthcare personnel then identified patients who might be interested in participating and who met the inclusion criteria, that is, being both male or female adults (18 years old or more of age), possessing Italian citizenship and, as regards participants’ clinical condition, being in need of a coronary angiography and angioplasty. These patient characteristics were the ones that participants were also asked about during the data collection phase of the research.

Moreover, the healthcare personnel also identified people who could not take part in the study because of the presence of exclusion criteria, that is, the presence of intellectual disability and thus a difficulty in properly understanding the study procedures and communicating. This evaluation was based on whether they were autonomous in understanding what they read and what was reported to them verbally.

The healthcare personnel, and more specifically, cardiologists of the hospital ward, selected patients who could participate in the research and approached them directly for recruitment.

Before starting the actual research procedure, each participant was asked to carefully read and sign an informed consent form that explained the purposes of the research and the methodology used, provided the researchers’ contact details in case participants had any questions concerning the study and clarified that all information on the patients would remain anonymous. The study followed the APA Ethical Principles of Psychologists, the Code of Conduct and the principles of the Declaration of Helsinki.

### 3.2. Data Collection and Analysis

Once all participants had provided informed consent, the control group was given a traditional paper-based informed consent for the procedure of coronary angiography, while the experimental group received an alternative multi-media informed consent. The informed consent for the experimental group consisted of a video lasting approximately 10–15 min, within which it was possible to see, in a virtual format, the steps and procedures involved in the coronary angiography. The video featured an actress who described the purposes of the examination, the therapeutic alternatives, the potential risks, the modalities of post-operative recovery, the types of results the diagnostic examination could return and the related therapeutic indications that could follow (such as continuation with pharmacological treatment, surgery, etc.).

The paper-based informed consent given to the control group was the version used in all Italian hospitals (with a few variations) and contained all the information deemed necessary to ensure the patient’s awareness of the procedure; the information provided was the same as that provided in the video-based informed consent.

Subsequently, before patients underwent the coronary angiography procedure, both groups were asked to fill out the same two questionnaires; the first one (created ad hoc by the researchers) was intended to assess the patient’s level of understanding of the content of the informed consent, the degree of perceived usefulness and the patient’s subjective perception of having understood the information provided, while the second one, the Depression Anxiety Stress Scale (DASS-21), assessed the patient’s levels of stress, anxiety and depression.

The first questionnaire consisted of nine items. The first seven items assessed the patient’s objective understanding of the procedure they were about to undergo by presenting questions concerning aspects of the coronary angiography to which the individual had to respond in a dichotomous way (yes or no). The following is an example item: “Is it possible to see the entire coronary tree with methods different from coronary angiography?”.

The eighth item examined the patient’s subjective evaluation of their level of understanding of the information provided (“How much do you believe you have understood the information presented in this document/video?”), and the final item explored the degree of usefulness the participants perceived the informed consent to have (“How much do you believe reading this document/seeing this video has been useful to you?”).

The items assessing participants’ subjective understanding of the information provided and the usefulness of the informed consent used a five-point Likert scale, where 1 corresponded to the evaluation “not at all” and 5 corresponded to the evaluation “completely”.

The second questionnaire provided was the DASS-21, a reduced version of the original self-report scale developed by Lovibond and Lovibond [41], which was created with the initial aim of providing maximum differentiation between the main symptoms of depression and anxiety. The updated version contains 21 items [42], divided equally into three scales that measure the levels of depression (evaluation of lack of incentives, low self-esteem and dysphoria), anxiety (referring to somatic and subjective symptoms of anxiety as well as acute fear responses) and stress (evaluating irritability, impatience, tension and persistent excitability). The validated Italian version was used for the present study, which, in terms of internal consistency, showed a Cronbach alpha of 0.87 for the depression dimension, 0.80 for the anxiety dimension, 0.89 for the stress dimension and 0.93 for the total general score [43]. It also showed high convergent and divergent validity coefficients (RS ranging from 0.50 to 0.80 and from −0.16 to −0.48, respectively) and good construct validity [43].

For the DASS-21, the subjects were asked to read each sentence and indicate how often (on a Likert scale from 0 to 3, where 0 = “it has never happened to me” and 3 = “it has almost always happened to me”) they have experienced a similar situation in the past seven days.

The data collected through the questionnaires were subsequently recorded in Excel and subjected to statistical analysis using the Statistical Product and Service Solution (SPSS) programme, version 17 (IBM, New York, NY, USA). Using the statistical programme, it was possible to carry out a comparison between groups; the programme’s tools were used to analyse and compare the results obtained in order to achieve the study objectives.

## 4. Results

Forty people took part in the present study, with an overall age ranging from 34 to 90 years (mean age = 68.55; SD = 13.03). The group consisted of 28 males (70% of the sample) and 12 females (30% of the sample), and 65% of the participants had never had a coronary angiography before, with 2.5% having had two or more.

Participants were randomly divided into two equal groups of 20 people: an experimental group, which received informed consent in a video-based format, and a control group, which received informed consent in the traditional paper-based format.

The members of the experimental group had an average age of 66.45 years (SD = 12.65), while the control group had an average age of 70.65 years (SD = 13.38). Therefore, there were no significant differences in age between the two groups. The experimental group consisted of 12 men and 8 women, while the control group consisted of 16 men and 4 women. Therefore, the difference between the two groups was minimal and not statistically significant, making the two groups homogeneous.

In terms of responses to the first questionnaire, significantly different results emerged between the experimental group and the control group. The experimental group obtained a mean score of correct answers in the first phase of the questionnaire (measuring objective understanding of the information provided) equal to 6.85 (SD = 0.37), while the control group obtained a mean score of 4.85 (SD = 1.14). Note that the maximum number of correct answers possible was 7.

Based on these results, it can be said that the experimental group—the one given the video-based informed consent—obtained on average a greater number of correct answers than the group given the traditional paper-based informed consent. This affirms that there was a statistically significant difference between the two groups in terms of responses to the first part of the questionnaire.

As for the subjective understanding perceived by both groups, measured on a Likert scale from 1 to 5, the experimental group obtained a mean score of 4.60 (SD = 0.60), while the control group obtained a subjective understanding mean score of 3.25 (SD = 1.41). Thus, a statistically significant difference was also noticeable between the two groups in this case, affirming that the experimental group had a greater subjective perception of their understanding of the information provided through the video-based informed consent than that of the control group.

The last part of the questionnaire concerned the perceived usefulness of the informed consent, and the responses were statistically different between the two groups in this aspect as well: the experimental group described the usefulness of the video-based consent with a mean score of 4.90 (SD = 0.31), while the control group described the usefulness of the paper-based consent with a mean score of 3.55 (SD = 0.83). These data suggest that the experimental group judged the instrument as particularly useful and more useful than the control group perceived the paper-based informed consent (Table 1).

For the second questionnaire, the DASS-21, which measured the levels of depression, anxiety and stress in the participants of the two groups, no statistically significant differences emerged between the data from the two groups. More specifically, for the dimension relating to depression, the experimental group obtained a mean score of 6.40 (SD = 5.62), while the control group obtained a mean score of 7.25 (SD = 5.55). For anxiety, the experimental group obtained a mean score of 9.00 (SD = 4.32), while the control group obtained a mean score of 9.65 (SD = 5.00), and for stress, the experimental group obtained a mean score of 12.10 (SD = 5.39), while the control group obtained a mean score of 10.50 (SD = 5.99). The mean scores related to general distress were 27.50 (SD = 13.10) for the experimental group and 27.40 (SD = 14.43) for the control group (Table 2).

In light of these results, it can be stated that there was no statistically significant difference between the levels of general distress, measured by DASS-21, between the two groups.

## 5. Discussion

The present research aimed to analyse the effectiveness of using video-based informed consent compared to using the more traditional paper-based informed consent with patients waiting to undergo coronary angiography.

The first of the more-specific objectives of the research was to verify the patients’ understanding of the information provided by comparing the results of the experimental group with those of the control. The results obtained in this regard showed a significant difference in favour of the experimental group, whose scores relating to the objective understanding of the information provided were greater than those of the control group. It is therefore possible to hypothesize that video-based informed consent is more likely to guarantee that patients have a greater understanding of the information provided and of the procedure they are going to face than a traditional paper-based informed consent.

The second specific objective of the study was to analyse patients’ perceptions of their own understanding of the information contained in the informed consent. The results in this case also showed the experimental group to perceive themselves as having a clearer understanding of the information received than that of the control group. These results suggest that video-based informed consent is better at making patients confident in their awareness and understanding of the procedure they are about to undergo than traditional paper-based informed consent.

The third objective of the research was to verify the perception of usefulness of the two different modes of informed consent. Once again, the experimental group reported a greater perception of the usefulness of the informed consent than the control group, thus allowing the hypothesis that patients perceive video-based informed consent as more useful than traditional paper-based consent.

Thus, from the obtained results, it can be stated that alternative formats of informed consent, specifically the presentation of the information necessary to the patient via video, can be more understandable both objectively and subjectively and therefore be perceived as more useful and effective by patients waiting to undergo invasive healthcare procedures such as coronary angiography.

These results appear to align with the findings of previous studies in the international literature from both the more general healthcare sector [14,16,17,18] and specifically in terms of cardiology and informed consent for coronary angiography [33,35,37,38]. These studies also found multi-media and video-based informed consent formats to be more effective at facilitating patients’ comprehension and recall of the fundamental information of the procedure and at providing greater confidence in the information delivered by these innovative instruments than traditional paper-based informed consent, which have significant limitations [7,8,11,29].

Lastly, the fourth objective of the study was to analyse the variables of anxiety, stress and depression in the two groups to evaluate whether video-based informed consent has an excessively intense psychological impact on patients by virtue of being more explicit than paper-based informed consent. The results of this study found no statistically significant differences between the scores related to levels of anxiety, stress and depression in the experimental group compared to those of the control group.

Although these psychological aspects are complex and linked to various variables in the patient’s life, the data from this study suggest that video-based informed consent does not present a greater risk to the patient’s mental state than traditional paper-based informed consent, indicating that there may be no particular or significant contraindications in terms of the patient’s mental health to implementing video-based informed consent.

This result also appears to align with recent literature in the field, with studies having found that a more detailed and engaging presentation of information does not lead to an increase in levels of anxiety or a worsening of mood states, instead finding that the more accurate information that can be provided via video may reduce the anxiety levels of people waiting for complex medical procedures, particularly surgical ones such as coronary angiography [37,38].

As has been previously indicated, the present study represents one of a very limited number of Italian contributions to the literature on multi-media informed consent in cardiology. It could therefore represent a starting point for future research in this area, considering that the results confirm those of other studies on the value and usefulness of alternative formats of informed consent, particularly video-based informed consent.

This study has some limitations, including the small sample size, which was limited to 40 participants because the study was implemented over the course of a few months.

However, even though the sample size is small, the authors believe the study results could still represent an important starting point for future research since very few studies have explored these variables and no studies had been previously conducted in this filed in Italy.

Related to this, another limitation is represented by the fact that, even though not statistically significant, the number of male and female participants could not be properly balanced, with the experimental group consisting of 12 men and 8 women and the control group consisting of 16 men and 4 women.

To ensure greater validity, future studies should therefore use a larger sample, as well as achieve a better balance between male and female participants, and be conducted over a longer time span to obtain wider results.

Moreover, the procedure of the present study did not allow verification of whether other variables of patients’ personal and clinical histories could have an impact on their perceived efficacy of the means of informed consent, since only a very contained number of variables has been taken into consideration for the present research. Therefore, future research should consider other relevant variables that could affect the results, such as per-existing clinical conditions or the patient’s level of education.

The study results are also limited by the exclusive focus on a single hospital and on patients in the cardiology department waiting to undergo coronary angiography; extending the use of video-based informed consent to other departments could determine whether its effectiveness can be significant in other contexts and with different kinds of patients. Finally, it would be very useful to know whether the same results can be obtained by other Italian hospitals using the same methodology.

Future studies should therefore address the limits still present in the traditional methods of informed consent, particularly where alternative methods for informed consent are still rarely applied or studied, such as in Italy. Such research could provoke changes that allow patients to obtain a more effective understanding of the proposed procedure, which is a key element to ensuring decision-making freedom, to overcoming the persistent paternalistic approach to medicine and to completing the move to patient-centred healthcare [44].

## 6. Conclusions

The results of the present study—that alternative formats of informed consent, such as video-based informed consent, are of greater general efficacy and usefulness and are more compatible with the patient’s well-being than traditional paper-based informed consent while not introducing risks to the patient’s mental state—can be considered elements of central importance for the future of cardiology and healthcare practices in general, representing a significant step towards the creation of healthcare that is effectively patient-centred in both intentions and actions.

## Figures and Tables

**Table 1:** Comparison between the experimental (EG) and the control group (CG) concerning objective and subjective understanding and utility perceived of the informed consent.

Measure	EG			CG			Test t		Mann–Whitney Test	
	M	SD	Med	M	SD	Med	t (38)	*p*-Value	U	*p*-Value
Questionnaire total score	6.85	0.37	7.00	4.85	1.14	4.50	7.49	<0.001	29	<0.001
Comprehension score	4.60	0.60	5.00	3.25	1.41	4.00	3.94	<0.001	83	0.001
Usefulness score	4.90	0.31	5.00	3.55	0.83	4.00	6.85	<0.001	31	<0.001

M = mean; SD = standard deviation; Med = median.

**Table 2:** Scores on the DASS-21 obtained by the experimental (EG) and the control group (CG).

Measure	EG			CG			Test t		Mann–Whitney Test	
	M	SD	Med	M	SD	Med	t (38)	*p*-Value	U	*p*-Value
dass_depression	6.40	5.62	6.00	7.25	5.55	7.00	−0.48	0.633	181	0.606
dass_anxiety	9.00	4.32	9.50	9.65	5.00	10.00	−0.44	0.662	183	0.635
dass_stress	12.10	5.39	12.00	10.50	5.99	11.50	0.89	0.380	167	0.371
dass_total	27.50	13.10	28.50	27.40	14.43	28.00	0.02	0.982	197	0.935

M = mean; SD = standard deviation; Med = median.

## Data Availability

Data are available upon reasonable request.
